# Reviewed article: Rocha VF, Barbosa MS, Neves E, Santos VJ, Rego RFS, Pessoa TL, Ribeiro MT, Santos JLA, Pereira FM, Reis JN. Vancomycin-resistant Enterococci infections in a hospital in Salvador, Bahia, Brazil: a descriptive study, 2021-2023. Epidemiol Serv Saude. 2025:34;20240135

**DOI:** 10.1590/S2237-96222025v34e20240135.a

**Published:** 2025-04-28

**Authors:** Osmar de Oliveira Cardoso

**Affiliations:** Universidade Federal do Piauí

**Table:** 

Rating	Excellent	Good	Average	Below average	Poor
Interest		✓			
Quality	✓				
Originality		✓			
Overall	✓				

**Table:** 

Questionnaire	Yes	No	Not applicable
Does the manuscript contain new and significant information to justify publication?	✓		
Does the Abstract (Summary) clearly and accurately describe the content of the article?	✓		
Is the problem significant and concisely stated?	✓		
Are the methods described comprehensively?	✓		
Are the interpretations and conclusions justified by the results?	✓		
Is adequate reference made to other work in the field?	✓		

## Manuscript Structure:

Length of article is: Adequate

Number of tables is: Adequatew

Number of figures is: Adequate

Please state any conflict(s) of interest that you have in relation to the review of this paper (state “none” if this is not applicable): None

## Comments

Minhas preocupações com o artigo são as seguintes:

1) A Figura 1 se apresenta confusa, com dificuldade para o entendimento do leitor, sugiro então o gráfico de barra seja alterado para um gráfico de linhas, já que certamente um caso teve impacto nos casos seguintes, e os leitores perceberiam melhor a situação em cada mês com a tipagem da cepa.

2) Na tabela 1 eu sugiro uma nova coluna com a porcentagem em separado e no final, na soma dos números absolutos, a somatória das porcentagens, devendo totalizar 100%.

3) Imagino que o trabalho será traduzido para o inglês, mas na versão para a revisão, o título do trabalho está em inglês e o corpo do texto em português, sugiro tb alteração para esse título.

